# Supplementary material for: Does the “Belt and Road Initiative” benefit the environment? Insight from analysis of intra-industry trade in environment goods
Source: PLoS One. 2024 Apr 2;19(4):e0300603. doi: 10.1371/journal.pone.0300603 (PMC10986933; doi:10.1371/journal.pone.0300603)
Supplement: Attachment — Submitted filename: PLOS ONE BRI.docx [file pone.0300603.s007.docx]

***Article***

**Does the “Belt and Road Initiative” Benefit the Environment? Insight from Analysis of**

**Intra-Industry Trade in Environmental Goods**

**Review**

The reasoning, concept, and hypothesis are strong and convincing. My comments are below

1. Overall good abstract but the author should add more scientific things in an abstract.
2. Why author choose this topic? What’s the novelty of your topic?

3. The author should discuss technical and scientific things in an abstract.

4. Where is your conceptual framework? Or Need of the study?

5. The author should improve the quality of words instead of the quantity of words.

6. The author should read the latest research papers and use them in the review of the literature.

7. The author should try to improve the methodology and discuss all the technical things in the methodology section.

8. How to verify results? What is the reliability of data and results?

9. The author should add a discussion section as well after the empirical results. And try to discuss all the empirical results properly one by one in the discussion section.

10. Try to be concise and to the point as much as possible.

11. I am not satisfied with the author’s citations because of a few mistakes in the citation. You should rewrite your references and try to use the latest literature.

12. Try to explain your points in an appropriate way.
