# Supplementary material for: Does the “Belt and Road Initiative” benefit the environment? Insight from analysis of intra-industry trade in environment goods
Source: PLoS One. 2024 Apr 2;19(4):e0300603. doi: 10.1371/journal.pone.0300603 (PMC10986933; doi:10.1371/journal.pone.0300603)
Supplement: Attachment — Submitted filename: Response to Reviewers.docx [file pone.0300603.s008.docx]

Dear Editor,

I hope this letter finds you well. I am writing to respond to each point raised by the academic editor and reviewers regarding the article titled "Does the "Belt and Road Initiative" Benefit the Environment? Insight from Analysis of Intra-Industry Trade in Environment Goods" (Manuscript Number: PONE-D-23-29967). This article is currently under consideration for publication in PLOS ONE. All the modifications in detail are as follows:

1. Overall good abstract but the author should add more scientific things in an abstract.

Respond: We had modified the abstract.

2. Why author choose this topic? What’s the novelty of your topic?

Respond: Although the BRI has always followed green and sustainable development principles, the BRI’s environmental impacts and its implications for environmental governance have been questioned in recent years. This is the main reason for which we choose this topic. The novelty of our topic is that we used the two-way fixed effect staggered differences in differences (TWFE Staggered DID) method to examine how the BRI impacts the EGs IIT between China and B&R countries, so as to study whether the BRI has positive impacts on environment.

3. The author should discuss technical and scientific things in an abstract.

Respond: We had modified the abstract.

4. Where is your conceptual framework? Or Need of the study?

Respond: We had added the conceptual framework.

5. The author should improve the quality of words instead of the quantity of words.

Respond: We had made another polish to the language of the whole article.

6. The author should read the latest research papers and use them in the review of the literature.

Respond: After careful searching, we found that we have used the latest literature related to this article.

7. The author should try to improve the methodology and discuss all the technical things in the methodology section.

Respond: We had added the section titled "Identification strategy" to give a clearer explanation of the method than before.

8. How to verify results? What is the reliability of data and results?

Respond: We will provide you with the original data and Stata code as a separate file.

9. The author should add a discussion section as well after the empirical results. And try to discuss all the empirical results properly one by one in the discussion section.

Respond: We had added a discussion section to discuss all the empirical results.

10. Try to be concise and to the point as much as possible.

Respond: We had tried to make all the languages as concise as possible.

11. I am not satisfied with the author’s citations because of a few mistakes in the citation. You should rewrite your references and try to use the latest literature.

Respond: We had solved the above problem.

12. Try to explain your points in an appropriate way.

Respond: We had modified it.

Thank you for your time and attention to this matter. I trust that PLOS ONE is committed to upholding the highest standards of scientific integrity. If you require any further information or have any questions, please feel free to contact me at [zhouyacheng@gmail.com](mailto:zhouyacheng@gmail.com) or [liufy@ctbu.edu.cn](mailto:liufy@ctbu.edu.cn).

I look forward to your positive response and appreciate your continued support.

Sincerely,

Weidong Huo

Professor

School of Finance and Trade, Liaoning University, Shenyang, China
